# Advanced Computational Methodologies Used in the Discovery of New Natural Anticancer Compounds

**DOI:** 10.3389/fphar.2021.702611

**Published:** 2021-08-17

**Authors:** Vivek P. Chavda, Yavuz Nuri Ertas, Vinayak Walhekar, Dharti Modh, Avani Doshi, Nirav Shah, Krishna Anand, Mahesh Chhabria

**Affiliations:** ^1^ Department of Pharmaceutics and Pharmaceutical Technology, L.M. College of Pharmacy, Ahmedabad, India; ^2^ Department of Biomedical Engineering, Erciyes University, Kayseri, Turkey; ^3^ ERNAM-Nanotechnology Research and Application Center, Erciyes University, Kayseri, Turkey; ^4^ Department of Medicinal Chemistry, Bharati Vidyapeeth’s Poona College of Pharmacy, Pune, India; ^5^ Department of Chemistry, SAL Institute of Pharmacy, Ahmedabad, India; ^6^ Department of Pharmaceutics, SAL Institute of Pharmacy, Ahmedabad, India; ^7^ Faculty of Health Sciences and National Health Laboratory Service, Department of Chemical Pathology, School of Pathology, University of the Free State, Bloemfontein, South Africa; ^8^ Department of Pharmaceutical Chemistry, L.M. College of Pharmacy, Ahmedabad, India

**Keywords:** natural anticancer compounds, theranostics, multi-target approach, fragment-based screening, drug antibody conjugate, drug repurposing, personalized medicine

## Abstract

Natural chemical compounds have been widely investigated for their programmed necrosis causing characteristics. One of the conventional methods for screening such compounds is the use of concentrated plant extracts without isolation of active moieties for understanding pharmacological activity. For the last two decades, modern medicine has relied mainly on the isolation and purification of one or two complicated active and isomeric compounds. The idea of multi-target drugs has advanced rapidly and impressively from an innovative model when first proposed in the early 2000s to one of the popular trends for drug development in 2021. Alternatively, fragment-based drug discovery is also explored in identifying target-based drug discovery for potent natural anticancer agents which is based on well-defined fragments opposite to use of naturally occurring mixtures. This review summarizes the current key advancements in natural anticancer compounds; computer-assisted/fragment-based structural elucidation and a multi-target approach for the exploration of natural compounds.

## Introduction

**FIGURE 1:**
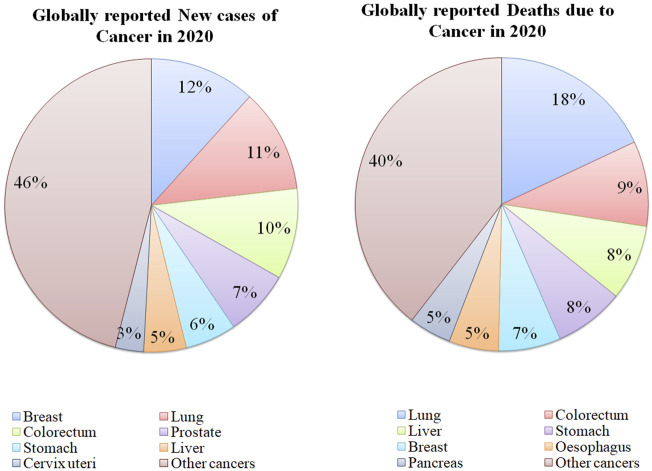
Statistics of globally reported cases and deaths due to various types of cancers in 2020 [Data Collected from (WHO, 2021a)].

Cancer is a disorder in which cells proliferate abnormally deprived of control. These cancerous cells can attack other nearby tissues and transfer to other body parts *via* lymph and blood. Numerous types of cancers exist such as carcinoma (cancer of the skin or internal organs covering the tissues), leukemia (cancer of tissue that forms blood like, bone marrow, which leads to the production of too many defected blood cells), sarcoma (cancer of muscle, bone, fat, cartilage, blood vessels or other connective tissues or supportive tissues), lymphoma and multiple myeloma (cancer of the immune system cells) (NIH, 2021). Healthy cells can change into tumor cells by following the multiple-stage processes resulting from the interaction between genetic factors of the person and external parameters. These external parameters include physical carcinogens, for example, UV and ionizing radiation, chemical carcinogens like asbestos, constituents of tobacco smoke, food contaminants (aflatoxin), drinking water contaminants (arsenic), and biological carcinogens such as infection of virus, bacteria, or parasites (Setlow, 2001). Globally, cancer is the second major reason for mortality with an estimate of 10 million deaths in 2020 (Figure 1) among which, breast, lung, colon, rectal, prostate, skin, and stomach cancers were the most frequently observed. Lung cancer was responsible for the majority of cancer-related deaths in 2020 (WHO, 2021b). Throughout the globe, about 1 out of 6 deaths occur because of cancer each year. In low and middle-income countries, almost 70% of the deaths arise due to cancer, out of which, one-third of fatalities are caused by consumption of tobacco and alcohol, high body mass index, less intake of fruits and vegetables, and insufficient physical activity (Wogan et al., 2004). Several infections including human papillomavirus, *H. pylori,* Epstein-Barr virus, hepatitis B virus*,* and hepatitis C virus (HBV and HCV) can also become indirect risk factors for cancer.

Advancements in multimodal imaging, 3D visual technology, combinational therapy, and the use of nanomedicine have improvized the effectiveness of cancer treatment in the past 2 decades (Shi et al., 2017; Chavda et al., 2019). Progress in cancer immunotherapy, chemotherapy, gene therapy, and epigenome therapy enables substantial improvement in cancer management. Apart from that surgeries including plastic surgery, robotic laparoscopy, radiotherapy, hormonal (mainly for breast and prostate cancer), and photothermal (laser) therapy has revolutionized cancer management (Kumar, 2018). The discovery of targeted drugs, monoclonal antibodies, checkpoint inhibitors, cancer vaccines (prophylactic and therapeutic), cytokines (interferon and interleukins), and chimeric antigen receptor T-cell (CAR-T cell) therapy is proven to be safe and effective in cancer management. Proton therapy is effective in the treatment of several cancer types such as brain, prostate, liver, lung, esophagus, breast, colon, eye, neck, and head. Advancements in robotic surgery enabled the treatment of certain cancers such as kidney, bladder, prostate, ovaries, throat, and uterus (Charmsaz et al., 2018). Many trials and research are ongoing in oncology for enhancing the efficacy of the treatment with minimum side effects.

Targeted drug delivery has proven effective in the treatment of various cancers with fewer side effects. Trastuzumab is used for breast cancers with human epidermal growth factor receptor 2 (HER-2) gene mutation. Afatinib and cetuximab block epidermal growth factor receptor (EGFR), which supports the development of colorectal and lung cancers. Dabrafenib and vemurafenib treat melanomas having mutated BRAF genes. Even though such targeted therapies are promising, currently they are used in very few types of cancers. Research towards improving such therapies is ongoing (Charmsaz et al., 2018). Multi-target therapy (either in sequential order or in combination) is also a widely used approach especially in the cases of intrinsic and acquired resistance towards anti-cancer compounds (Holohan et al., 2013). Drugs such as sunitinib, sorafenib, vandetanib, pazopanib, and axitinib are examples of multi-target treatment of cancer. Sunitinib is approved to treat gastrointestinal stromal tumors and renal cancer. Similarly, sorafenib is approved for renal cancer and liver cancer, where both drugs have the potential to target multiple sites. Vandetanib, pazopanib, and axitinib are used for treating non-small-cell lung cancer (NSCLC) and breast cancer. Vandetanib is effective in thyroid cancer, pazopanib in ovarian and kidney cancer, and axitinib in renal and pancreatic cancer (Petrelli and Valabrega, 2009).

Approximately 60% of effective anticancer drugs are obtained from natural sources (Newman and Cragg, 2012). Campothesins, nucleosides, taxanes, and vinca alkaloids are widely used anticancer drugs obtained from natural origin, and these can provide potential clinical efficiency with diminished toxicity. Another important chemotherapeutic anticancer role is offered by combining potent cytotoxic natural compounds with monoclonal antibodies, particularly targeting antigenic determinant sites of tumors (Table 1) (Cragg and Pezzuto, 2016). Chemo-preventive and tumor-suppressive activities exerted by certain natural agents such as resveratrol, curcumin, indole-3-carbinol, (-)-epigallocatechin-3-gallate, and vitamin D have been reported in multiple studies (Weng et al., 2008; Chakraborti, 2011; Ko et al., 2017; Kwak et al., 2017; Tomeh et al., 2019).

**TABLE 1:** Pharmacological and Pharmacognostic details of the naturally occurring anticancer compounds.

Active chemical constituents	Plant name and (plant family)	Mechanism of action	Biological target	References
Shikonin	*Lithospermum erythrorhizon* Siebold and Zucc. (Boraginaceae)	Cell proliferation and cell death	Inhibition of CDC25 and CDK1	Zhang et al. (2019)
Columbianadin	Angelica decursiva (Miq.) Franch. & Sav. (Apiaceae or Umbelliferae)	Angiogenesis	Inhibition of VEGF,EGF,PDGF, TNF-α	Majnooni et al. (2019)
Polyphyllin D	*Paris polyphylla* Sm. (Melanthiaceae)	Apoptosis	Inducing DNA fragmentation and phosphatidyl-serine (PS) externalization	Cheung et al. (2005)
Tanshinol A	*Salviae miltiorrhizae* Bunge (Lamiaceae)	Inducing autophagy and apoptosis and inhibiting cell growth and migration	Activating AMPK and inhibiting PI3K/Akt/mTOR signaling pathway	Tay et al. (2019)
Artesunate	*Artemisia annua* L. (Asteraceae)	Apoptosis, arrest of the cell cycle at G_0_/G_1_, and oxidative stress	Regulating the pathway of NK-B, survivin, NOXA, hypoxia-inducible factor-1α, and BMI-1	Das (2015)
Dihydroartemisinin	*Artemisia annua* L. (Asteraceae)	Inhibition of tumor hypoxia	Altering the ROS- dependent apoptosis which summarises the activation of pro-apoptotic Bcl-2 family member Bax, and caspase-activation	Ontikatze et al. (2014)
Phenethyl isothiocyan	*Cruciferous vegetables* (Cruciferouceae)	Inhibition of the progression of tumorigenesis	Pathway alteration of Akt, JNK, XIAP, MCl1, BCL2, BCL-XL, BAD, BAX	Gupta et al. (2014)
Piperlongumine	*Piper longum* L. (Piperaceae)	Cell cycle arrest, inhibition of angiogenesis, metastasis pathways, and autophagy pathways	Key regulatory proteins, including PI3K, AKT, mTOR, NF-kβ, STATs, and cyclin D1	Tripathi and Biswal (2020)
Metformin	*Galega officinalis* L. (Fabaceae or Leguminosae)	Inhibition of tumor development	Triggering AMPK pathway	Aljofan and Riethmacher (2019)
Gossypol	*genus Gossypium* (Malvaceae)	Inhibition of tumor necrosis	Inhibition of TNF- α and NF-κB	Lu et al. (2017)
Anthocyanin	*Brassica oleracea var* (Brassicaceae)	Suppression of angiogenesis	Inhibition of TNF- α, inducing VEGF expression	Wang and Stoner (2008)
Paclitaxel	*Taxus brevifolia* Nutt. (Taxaceae)	Arrest cells in the G2/M phase of the cell cycle	Inhibition of EGFR	Barbuti and Chen (2015)
Curcumin	*Curcuma longa* L*.* (Zingiberaceae)	Inducing apoptosis and inhibiting proliferation	Strong inhibition of TNF-α	Barbuti and Chen (2015)
Dimethoxy curcumin	*Curcuma longa* L. (Zingiberaceae)	Anti-tumor effect	Inhibition of EGFR, epithelial-mesenchymal transition, and VEGFR	Chen et al. (2016)
Curcuminoid B63	*Curcuma longa* L. (Zingiberaceae)	Inducing cell proptosis	Targeted TrxR1 protein and increases (ROS) level which responsible for MAPK pathway activation.	Chen et al. (2019)
Celastrol	*Tripterygium wilfordii* Hook.f. (Celastraceae)	Suppressing the development and progression of tumor	Multiple signaling pathways inhibition such as reactive oxygen species (ROS)/JNK and Akt/mTOR, NF-κb, STAT3/JAK2, HSP90, Cdc37, p23, Iκκb, p-Akt, ERα	Shi et al. (2020)
Ginsenoside Rh2	*Panax spp* (Araliaceae)	Inhibition of cell proliferation, cell cycle, cell invasion, and metastasis	Signaling pathway inhibition Akt/mTOR, NF-κb, STAT3	Li et al. (2020)
Hesperidin	Citrus aurantium*L* (Rutaceae)	Responsible for autophagy and apoptosis cell death,	The regulatory protein of Caspase3 and Aurora-A kinase	Korga et al. (2019)
γ-tocotrienol, delta- tocotrienol	*Elaeis guineensis* Jacq. (Arecaceae)	Inhibition of angiogenesis	Downregulation of NF-kappa B pathway and VEGF	Ling et al. (2012)
Withaferin A	Withania somnifera (L.) Dunal (Solanaceae)	Inhibition of angiogenesis and inducing intratumoral apoptosis	Encouraging the expression of pro-apoptotic protein Bax and NF-κB pathway inhibiting by caspase-3 protein	Lee and Choi (2016)
6-Shogaol	*Zingiber officinale* Roscoe (Zingiberaceae)	Inducing apoptosis	Blocking of NF-κB	Ling et al. (2010)
Berberine	*Berberis aetnesis* aetnensis C.Presl (Berberidaceae)	Inhibition of cell proliferation	Suppressing EMT and downregulating signaling pathways (ROS, inhibiting mTOR and Akt phosphorylation, AMPK)	Wang et al. (2020)
Honokiol	*Magnolia virginiana* L. (Magnoliaceae)	Regulation of cellular homeostasis	Downregulating signaling pathway AMPK/mTOR	Lee et al. (2019)
Sanguinarine	*Argemone mexicana* L. (Papaveraceae)	Inducing apoptosis and programmed cell death	Inhibition of Bax and Bcl-2 proteins	Xu et al. (2012)

Abbreviations: CDC, Complement-dependent cytotoxicity; CDK, Cyclin-dependent kinase; VEGF, Vascular endothelial growth factor; EGF, Endothelial growth factor; PDGF, Platelet-derived growth factor; TNF-α, Tumor necrosis factor-alpha; AMPK, AMP-activated protein kinase; mTOR, The mechanistic target of rapamycin; DNA, Deoxyribonucleic acid; BMI-1, Polycomb complex protein; ROS, Reactive oxygen species; Akt, Protein kinase B; JNK, c-Jun N-terminal kinases; STATs, signal transducer and activator of transcription proteins; MAPK, mitogen-activated protein kinase; NF—κB, nuclear factor kappa-light-chain-enhancer of activated B cells.

Some natural products are also used to prevent severe side effects of chemotoxic agents which include nausea, vomiting, loss of appetite, diarrhea, constipation, fatigue, skin irritation, etc. Hence, the natural compounds obtained from plants, marine sources, bacteria, fungi, and animals have great potential to effectively target carcinogenic cells with minimal side effects. In this review, we discuss the potential of phytochemicals as anticancer agents followed by new computational approaches like multi-target and fragment-based approaches for the natural anticancer discovery and at last, we discuss theranostic role of natural anticancer agents.

## Natural Anti-Cancer Compounds and Current Research

Having extraordinary diversity in nature, the plant-derived compounds are recognized as rich sources of bioactives with some of them also possesses theranostic potential. In the past four decades, many efforts have been made to isolate new chemical entities (NCE) from natural sources like plants, marine, and microorganisms to develop anti-cancer agents. Since 1981, around 25% of new anticancer molecules are derived from natural sources but most of the targeted small molecules were launched after the year 1990 (Beck et al., 2017). Different approaches for the development of novel natural anticancer drugs are summarized in Table 2.

**a. Antibody-Drug conjugates**

**TABLE 2:** Novel natural anticancer drug development approaches.

Methods	Natural ligands	Targeted PDB^a^	Activity	Analytical tools/Methodology	References
Structure-based and Ligand-based	Porphyrin derivatives	Interaction with Bcl2 active site (PDB: 1XJ)	Antitumor activity	Molecular dynamics (MD), Structure-based pharmacophore modeling, Molecular simulation (MS)	Arba et al. (2018)
	Curcuminoids, thiotryptophan, and 4-phenoxyphenol derivative	PDB of EGFR, MMP-9, mTOR, PKC AKR1B10 (PDB:1ZUA)	Antitumor activity	Molecular docking	Parsai et al. (2014)
	Pheophytin [high affinity human mitochondrial translocator protein (TSPO) ligand]	Inhibit mitochondrial membrane Potential in adenocarcinoma A549 cells	Cell survival	Molecular docking	Shailaja et al. (2019)
	4-Methylpteridinones (berberin)	PI3K/mTOR (PDB: 3OAW)	Cell survival	Molecular dynamics	Liu et al. (2010)
Fragment-based	Trypanosomabrucei, Trypanosomacruzi, Leishmaniainfantum, and Plasmodium falciparum.	Thioredoxin peroxidase 2 (Trx-Px2)	Antitumor activity	Molecular docking	Boucher et al. (2006)
Drug repositioning and purposing based	Metformin and Aspirin	*In-Vivo* cell line MCF-7, VEGFR-2 (PDB: 3ewz)	Inhibition of breast cancer cells	Molecular dynamics and Molecular simulation	Amaral et al. (2018)
	Mushrooms	Inhibition of Histone deacetylase (HDAC) (PDB: 3C10)	Inhibition of breast cancer cells (MCF-7 cell line inhibition)	Molecular docking	Maruca et al. (2020)
Antibody-drug conjugation	Gemtuzumab ozogmicin	Calichemicin monoclonal antibody	Inhibition of cytotoxic tumor	Disulfide-thiol exchange	Mullard (2014b)
Molecularly targeted drug	Hematoxylin analogs	Protein tyrosine kinase inhibitor (VEGFR-r PDB:4ASD)	Inhibition of angiogenesis	Molecular dynamics and molecular simulation	Ortiz-Hidalgo and Pina-Oviedo (2019)
Leveraging cutting-edge technologies	Withaferin A	Cysteine 377	Inhibition of breast cancer	*In silico* approaches	Sivasankarapillai et al. (2020)

a PDB: Protein Data Bank

The development of antibody-drug conjugation (ADCs) therapy in the early 1990’s that integrates monoclonal antibody (mAb) and potent chemotherapeutic agents in a single chemical moiety by a chemical linker has paved the way for the next-generation cancer therapeutics. This advanced approach explores targeting a mAb to improve tumor-specific drug delivery by the antigen-antibody interaction with enhanced anti-cancer activity. Physical and biological properties that are necessary for the ADCs are as follows: 1) Drug loading requires a suitable modified site for conjugation with mAb, 2) Appropriate solubility in water is crucial for desired antibody reaction, and 3) Considerably, higher toxicity (IC_50_ between 0.01 and 0.1 nM) than the standard chemotherapeutic agents is required (Abdollahpour-Alitappeh et al., 2019). Gemtuzumab ozogamicin was the first approved conjugate of humanized anti-CD33 monoclonal antibody which is covalently attached to the cytotoxic antitumor antibiotic calicheamicin, but it was withdrawn from the market in 2010 due to fatal adverse events like hemorrhage, infection, and/or acute respiratory distress syndrome. In 2011, brentuximab vedotin was approved for treating Hodgkin lymphoma and anaplastic large cell lymphoma (Mullard, 2014a).

**b. Structure-based drug design for natural products**

The structure-based drug design is a new approach that can be applied to naturally occurring molecules for the discovery of new anticancer agents. The application of such approaches, resulted in a substantial compilation of natural remedies with potential therapeutic activities against cancer which, while mostly immature as drug candidates, provide highly heterogeneous substrates for lead compounds. This is the most reliable approach for natural lead development. Hematoxylin and its analogs sourced from the heartwood of *Haematoxylon campechianum* L. manifested tyrosine kinase inhibitory potential (Lin et al., 2008).

**c. Drug mechanism-based evaluation of novel natural anticancer**

Cutting-edge technologies incorporated with chemoproteomics and multi-omics help to overcome challenges in the mechanistic investigation of naturally occurring drugs. Grossman et al. reported the use of chemoproteomics for the discovery of anti-cancer natural product withaferin A that targets cysteine 377 on the regulatory subunit PPP2R1A of the tumor suppressor protein phosphatase 2A complex, and impair breast cancer cell proliferation (Ward et al., 2019).

**d. Drug repositioning and repurposing**

**FIGURE 2:**
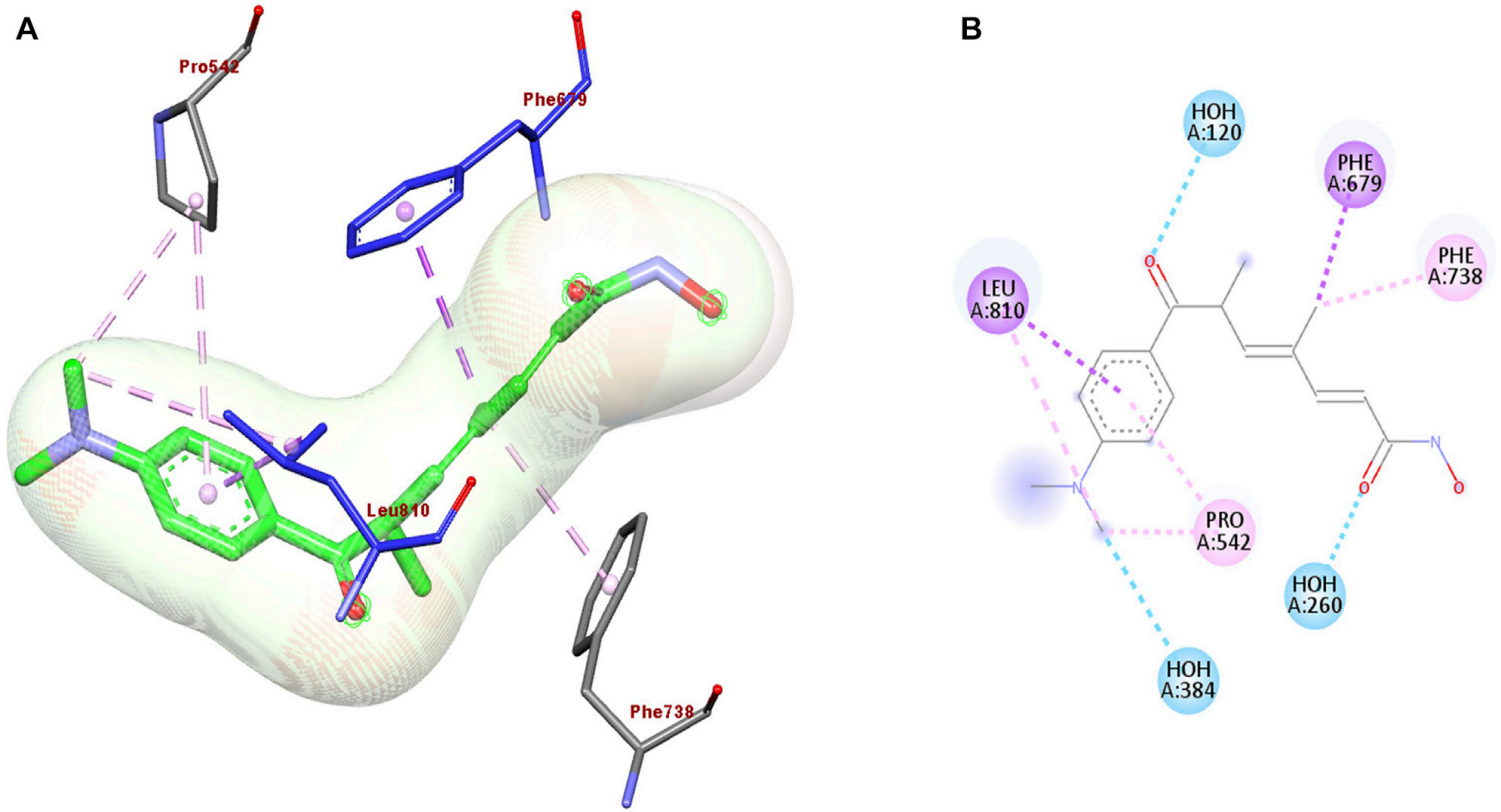
**(A)** 3D and **(B)** 2D representation of the best re-docking pose of Trichostatin A (TSA) against HDAC7 (PDB code 3C10) receptor obtained using the Glide-SP algorithm.

The discovery of novel anticancer agents from natural sources remains a challenging task. Therefore, many of the currently used natural anticancer drugs are derived from already existing drugs used for the treatment of different diseases as a part of drug repurposing. Drug repositioning approaches include structure-based and ligand-based drug designs. Mushrooms display antifungal, antimicrobial, antiviral, antitumor, and antioxidant activities. The antifungal activity of the mushrooms also inhibits histone deacetylase (HDAC) resulting in anti-proliferative activity against human breast cancer cell line MCF-7. As demonstrated in Figure 2, Trichostatin A docking with HDAC 7 crystal structure of the protein (PDB: 3C10) shows interaction with LEU 810, PRO 542, PHE 738, PHE 679, HOH 260, and HOH 384 aminoacids by hydrogen and π-π bond interaction (Maruca et al., 2020). Trichostatin A inhibits HDAC, resulting in anti-proliferative activity against the human breast cancer cell line, MCF-7.

## Computer Assisted Structural Elucidation of Natural Anticancer Compounds and Other Bioinformatics Approaches

The isolation and identification of natural compounds is a tedious task for chemists because natural compounds have more than one stereo-center, high molecular weight, and complicated chemical scaffolds (Koos et al., 2020). CASE strategies that are dependent on chromatographic and spectroscopic approaches are recently explored for structural elucidation of natural anticancer compounds (Elyashberg and Argyropoulos, 2020). However, to date, only a few spectral data are available for natural compounds, therefore structure elucidation remains a challenge. The spectroscopic and crystallographic analysis techniques accelerated the process of structural elucidation of natural molecules and helped in broadening the spectrum of structural elucidation that could be applied as a tool in the discovery of new drug entities (Nugroho and Morita, 2019).

**FIGURE 3:**
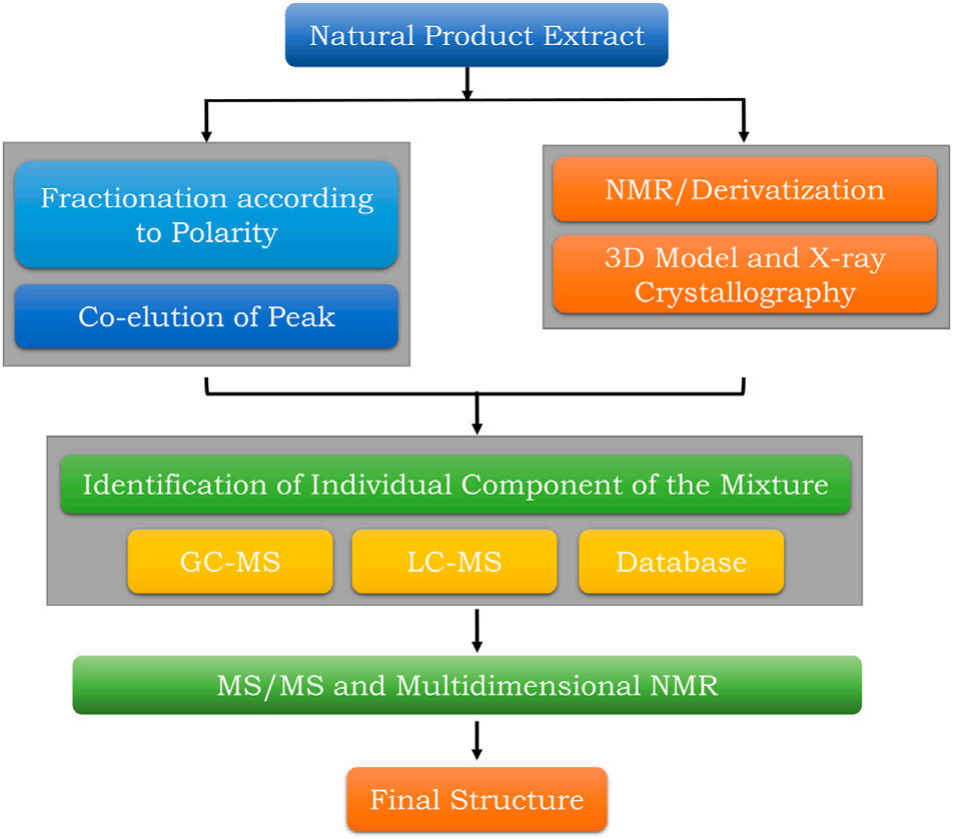
Summary of stepwise structural elucidation techniques of natural products.

Numerous advances in Mass Spectrometry (MS) and Nuclear Magnetic Resonance (NMR) over the last 2 decades have enabled structural elucidation processes for complex natural product mixtures (Figure 3). Qualitative analysis like MS gives information about the molecular weight and fragments of the sample. Recent scenarios of hyphenated techniques like LC-MS, GC-MS, GC-FID, GC-MSMS, and LC-MSMS are used for high-resolution molecular weight determination with a decrease in the total number of overlapping m/z ratios (Rinschen et al., 2019). Orthodox chromatographic approaches take a long time to identify or forecast the key chemical components found in a natural product mixture. The restricted compound references often render identifying or predicting each constituent in a mixture quite troublesome. As a result, an effective algorithm for solving the symmetric cone complementarity problem (CSCCP) is needed. Similarly, sensitive techniques like NMR spectroscopy give an essence of type and number of protons (H^1^), carbon (C^13^), phosphorous (P^31^), and fluorine (F^19^) content in the natural organic compounds. NMR spectrometric analysis alone cannot elucidate the structure of the natural organic compounds independently, therefore hyphenated techniques namely LC–NMR–MS and LC–UV–solid-phase extraction–NMR–MS are used. An IR spectrum can tell a lot about the existence or absence of such functional groups, but it does not give information about their environment in the compound (Elyashberg et al., 2009). It was suggested that 2D NMR data can be routinely generated, even in automation and a multitude of data are available as inputs to CASE systems like HSQC (HMQC), ^1^H-^1^H COSY (TOCSY), and HMBC methods (Elyashberg et al., 2009). Nowadays, 2D NMR spectroscopy is used for the structural elucidation and verification of natural organic molecules (Soong et al., 2020). A logical review of 2D NMR data often reveals the existence of “nonstandard” duration of COSY and HMBC correlations. Fuzzy structure generation allows for the right solution even though an uncertain number of nonstandard associations of unknown duration is present in the spectra (Su et al., 2017).

A new computer-aided software engineering (CASE) algorithm, known as the NP-Structure predictor, predicts individual components in a natural product mixture by using information acquired from LC-MS experiments (Harn et al., 2017). This is accomplished by comparing a list of known scaffolds with a list of weighted side groups to generate a list of potential molecules subject to defined structural constraints. They also mentioned an iterative DP algorithm with a wide range of potential sets of positions (Nk) that can be connected by the side chains upon its seed scaffolds, which may result in a challenging execution period for the algorithm (Kind and Fiehn, 2010; Harn et al., 2017). Expert system structure elucidator for the natural organic anticancer compound includes molecular connectivity diagram, structure generation, and verification and selection of the most probable structure. The fuzzy logic structure elucidator, which is based on spectral data, can easily identify large and complicated molecules consisting of up to 100 or more atoms with topological conceit. The beneficial predictions produced by computational models combined with empirical validations could help to accelerate the production of anti-cancer drugs from natural origin.

## Fragment-Based Screening of Natural Anticancer Compounds

**FIGURE 4:**
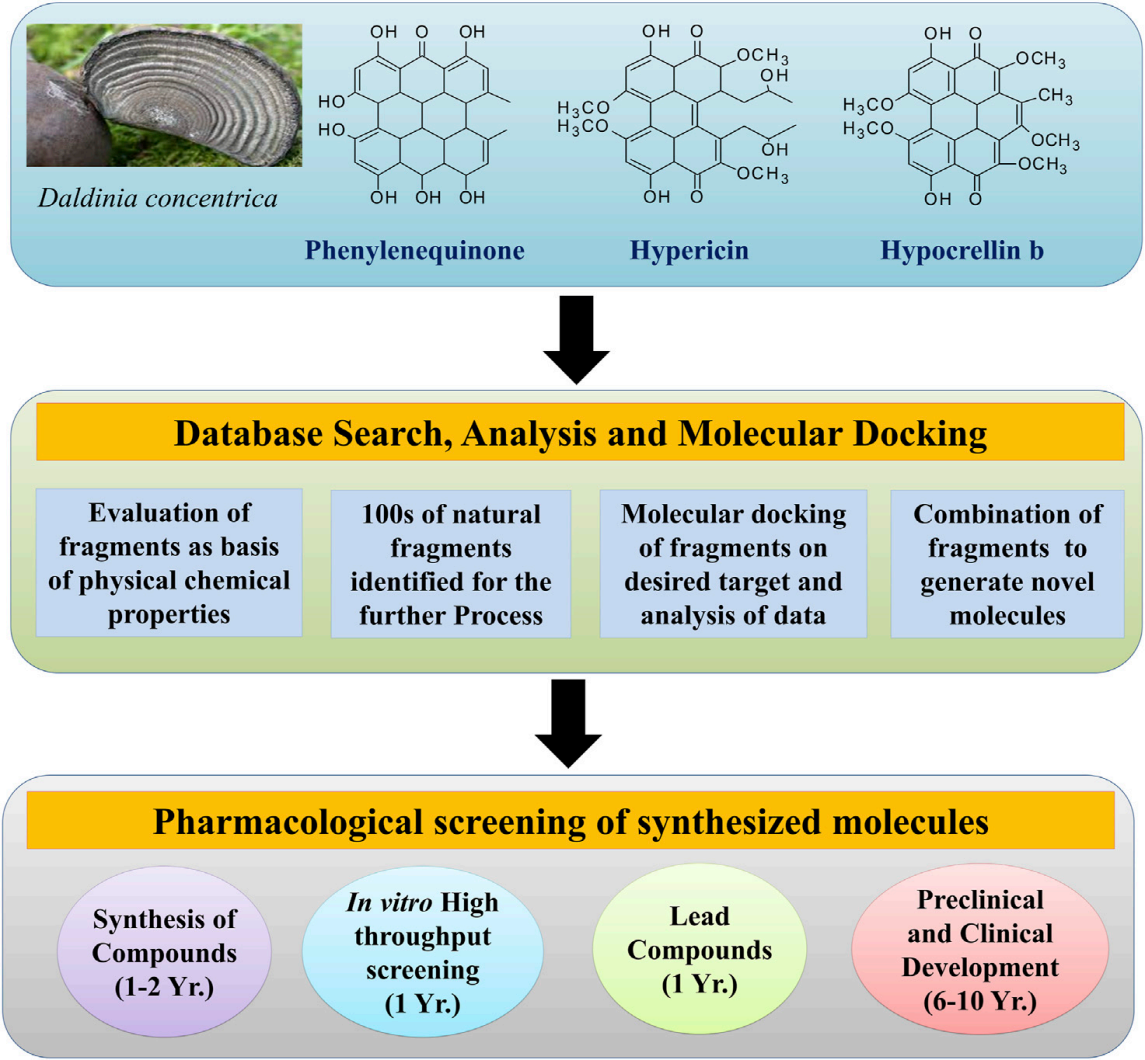
Procedure for fragment-based screening.

Fragment-based drug discovery (FBDD) will have wide applications in the field of natural products in the upcoming years because extraction, isolation, and purification of the active constituents from the natural sources are quite tedious and time-consuming (Khalifa et al., 2019). Application of *in-silico* techniques may expedite the process of development of potent and semisynthetic compounds that were originally isolated from natural sources. FBDD of the natural compounds is challenging because it is not yet largely explored for natural compounds manifesting anticancer activity (Murray and Rees, 2009). The natural chemical compounds that exhibit anticancer activity include alkaloids, polyphenolic compounds, etc. FBDD mainly depends on the molecular modeling strategy for the identification of the potential fragment for the anticancer agent. This involves the binding of the small fragments in the active site of the target (protein) to analyze the interaction of the small fragments with the target protein that helps the medicinal chemists and pharmacologists to design novel anticancer molecules. Natural anticancer compounds are sourced from plants, animals, and marine sources. The fragments are analyzed by various spectroscopic techniques and crystallographic techniques (David et al., 2020). Fragment-based screening (Figure 4) (Li, 2020) involves the following steps:

1. Collection of the compounds from the database
2. Filtration of the compounds from the database
3. Spectroscopic and crystallographic analysis of fragments
4. Molecular docking of the identified small fragments
5. Anti-cancer screening of the fragments.

Vemurafenib was the first drug discovered by the FBDD approach that consists of pyrollopyridine as a fragment for anticancer activity. Similarly, there is ample potential for various natural compounds to be screened as novel anticancer agents by applying the FBDD approach (Liu and Quinn, 2019). If a target has been established, bioinformatics can be used to comprehend the structure, which can be achieved by X-ray crystallography or other approaches such as homology modeling. The target protein would then be overexpressed. If the isotopically labeled protein is quickly purified and exhibits scattered cross-peaks in the 1H-15N-HSQC spectrum, this protein-based NMR can be used in screening. Otherwise, fragment scanning can be performed using DSF or 19F-NMR. If the target can be conveniently crystallized, X-ray crystallography would be the first tool to be used in screening. Where a target framework is open, the virtual screening will still be performed. An appropriate library would be chosen from a large number of sources, which is not a restriction. Structural, biophysical, and biochemical approaches can be used to validate the hits. Finally, appropriate techniques can be used to exploit fragment development (Figure 4). Role of medicinal chemists would be crucial in this phase.

Curcumin is an active constituent of *Curcuma longa* L. (Family: Zingiberaceae), which exhibits anticancer activity. It consists of the active fragment of β-diketone. Curcumin’s anticancer activities are due to its direct or indirect control of signal transduction pathways, *via* its effect on cell division, the p53 tumor suppressor gene, and transcription factors Nrf2 and NFB, as well as modulation of inflammatory signaling cascades. Similarly, for the designing of novel COX-2 inhibitors like diketone, is a pharmacophore for developing anticancer agent (Kljun and Turel, 2017).

Potential natural anticancer compounds which act on p21-activated kinase (PAK1) were reported by using computational methodologies (Shahinozzaman et al., 2020). 42 fragments of herbal compounds were identified from drugs such as triptolide, cucurbitacin I (C-I), nymphaeol A (NA), and staurosporine (SPN). Pharmacokinetic properties like absorption, distribution, metabolism, and excretion (ADMET) and molecular docking studies suggested that inhibitors C-I, NA, and SPN fit in the catalytic region of p21 activated kinase with promising pharmacological and pharmacokinetic parameters (Shahinozzaman et al., 2020). Molecular dynamic simulation studies revealed that NA shows tight binding with the PAK1 enzyme and can be considered to be safe while toxicity was manifested by SPN and C-I (Abdollahpour-Alitappeh et al., 2019).

AlAjmi et al. identified novel natural molecules as polo-like kinase (PLK-1) inhibitors with an anticancer activity using a computational modeling approach like FBDD (AlAjmi et al., 2018). Selleck’s library of natural compounds was screened against PLK-1 with the aid of a molecular docking approach. Docking studies identified eight bioactive natural molecules (Apigenine, Dihydromyrecetion, Hesperidin, Hesperitine, Naringenin, Phlorizi, and Quesertine) as PLK-1 inhibitors. Molecular Mechanics-Generalized Born Surface Area (MM-GBSA) calculations showed that hesperidin was found to be a potent inhibitor of PLK-1 by the formation of a sturdy complex with Tyrosine-protein kinase (TLK) and confirmed with the help of molecular dynamics simulation studies. The generated data provide ample evidence and confirmed that hesperidin is a potential PLK-1 inhibitor by following parameters as molecular weight (610.56 g/mol), 8 H-bond donors, 15 H-bond acceptors, 234 Å TpSA, 0 net charges, and 7 rotatable bonds. These results were more significant as compared to other natural inhibitors of PLK-1 as an anti-cancer agent. Similarly, optimization of natural compound Itampoli A as p38α inhibitor by application of FBDD approach was reported elsewhere (Liang et al., 2019). Itampoli A which is effective against lung cancer was isolated from *Iotrochota purpurea,* a marine sponge. Itampoli was modified with the aid of the FBDD strategy. A total of 45 brominated tyramine analogs were synthesized as fragments. *In-vitro* enzyme inhibition assay of 45 analogs was performed against the p38α enzyme. The inhibitory study revealed that (−)-itampolin A potentially inhibited p38α with an IC50 value of 7.9 ± 1.7 nM. (−)-itampolin A also inhibited cell proliferation in the lung cancer cell line (A549) at a concentration of 0.66 mM. Molecular docking revealed that (−)-itampolin A fit in the active site of p38α by forming 3H-bonds with GLU71 and ASP168. On the other hand, the 3D QSAR approach revealed that the tyrosine skeleton was essential for the p38α inhibitory activity which was mainly contributed by the ureoid moiety in (−)-itampolin A (Liang et al., 2019).

Therefore, FBDD is one of the attractive tools for the medicinal scientists in order to explore natural anticancer agents and further identify hit fragments from the reported natural anticancer agents and model a new anticancer molecule (Koos et al., 2020).

## Multi-Target Approach for the Exploration of Natural Anticancer Compounds

**FIGURE 5:**
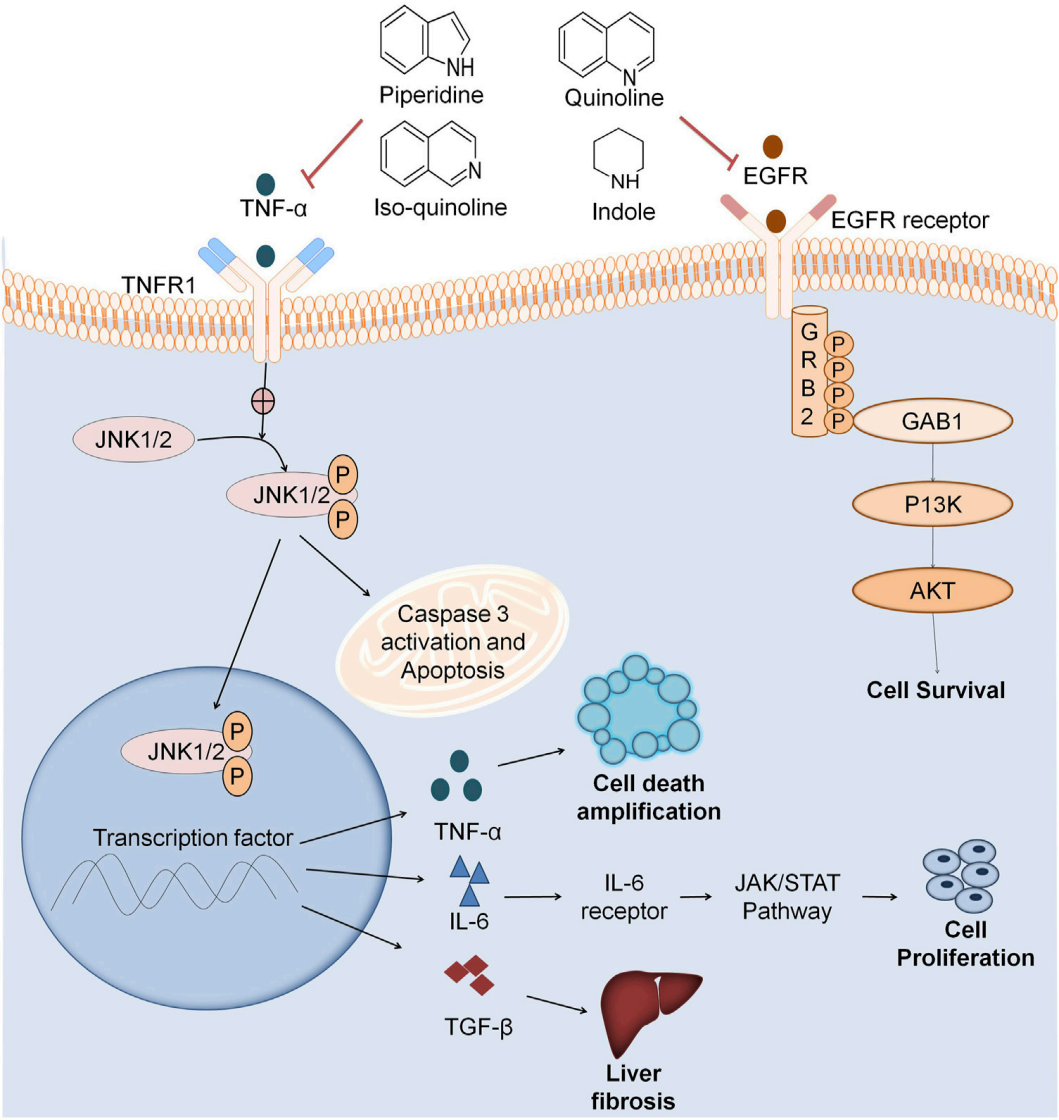
Multitarget based screening of anticancer natural compounds. Pro-inflammatory mediators like TNFα, JL2, JL12, etc. binds to the receptor on the cell surface which undergoes a conformational change and activates JNK 1/2. JNK1/2 phosphorylation leads to apoptosis that releases inflammatory factors like NFKβ that also releases inflammatory factors like TNF-α which plays role in cell death amplification. IL6 activates JAK/STAT pathway that results in cancer cell proliferation. TGFFβ also activates various pathways leading to liver fibrosis. EGFR binds to the cell surface receptor that activates GRB2. Phosphorylation of GRB2 activates the downstream pathway as GABI further activates P13K that activates AKT leading to cell survival. Natural fragments like indole, quinoline, isoquinoline, and piperidine inhibit both the inflammation pathway and EGFR in cancer cell growth.

The conventional drug discovery approach was applied for the discovery of novel drugs. Even until 2000, scientists developed drugs separately for different targets of the same disease. This approach leads to an increase in the number of drugs and their corresponding side effects (Table 3). To avoid this, scientists focused on developing drugs that can target multiple sites of the same disease that can ultimately reduce the number of drugs in the chemical space and also the corresponding cost to develop them (Figure 5) (Ramsay et al., 2018). The blockbuster drug Sorafenib was developed by Bayers and Onyx which gained approval from USFDA in 2007. Sorafenib inhibited different kinases namely VEGFR-2, VEGFR-3, PDGFRβ, c-Kit, and Raf, that play a crucial role in cancer progression and angiogenesis at a low nanomolar concentration. Similarly, in Indian traditional medicine, curcumin is a natural active constituent of crude drug turmeric and its semisynthetic analogs are used in the treatment of several types of cancers. In the last 3–4 decades, various drugs were developed which act by binding to diverse biological targets, resulting in desired pharmacological activity (Cragg and Pezzuto, 2016).

**TABLE 3:** Multitarget-based anti-cancer natural compounds.

Nature molecule/Crude drug	Inhibitory effect on biological targets or cancer cell lines	References
Anthracycline	MCF-7 and T47D	Cragg and Pezzuto (2016)
Pyrano-quinolone	COX-2 and EGFR signalling	Kumar and Adki (2018)
Taxol and Vinblastin	PI3K, APC, and RB signalling	Taylor et al. (2019)
Deacetylnemorone	SKMEL5 (melanoma cancer cells), MG-63 (osteosarcoma), SK-OV-3 (ovarian adenocarcinoma), MDA-MB-231 (breast cancer), HCT 116 (colorectal carcinoma), HCT 116/200 (FdUrd resistant subclone of HCT 116 cells), A2780ADR (a doxorubicin-resistant subclone of the ovarian carcinoma A2780), and HUVEC (Normal human umbilical vein endothelial cells).	Liu and Quinn (2019)
Curcumin	NF-κB, miR-221, COX-2, and their effectors such as PTEN, p27, p57, and pro-inflammatory cytokines. STAT-1, STAT-3 phosphorylation, and Notch signaling pathway. Pancreatic cancer cell lines (MiaPaCa-2, Panc-1, AsPC-1, BxPC-3, and Pan02)	Sahebkar (2016)
Curcumin	COX-2, STAT-1 and STAT-3 signaling, NF-κB, VEFG, EGFR signaling, PI3/Akt, and m-TOR signaling, CDK, B-catenine, Tcf-4	Sahebkar (2016)
Piperine	Nuclear factor-κB (NF-κB), c-Fos, CREB, ATF-2, Melanoma cell line (B16F-10 piperine concentration = 2.5, 5, and 10 μg/ml)	Pradeep and Kuttan (2004)

There are various targets for the treatment of different diseases like tyrosine kinase for cancer, cyclooxygenases (COX) for inflammation, and DNA gyrase for bacterial infection and tuberculosis. Marine plants are the main source of anticancer agents, and they can target more than one disease. Methanolic extract of *Artemia salina* is a marine brine shrimp that shows both anticancer as well as antibacterial activity. Natural antioxidants like selenium, vitamin E, myricetin, quercetin, and kaempferol act as anticancer agents *via* free radicals such as superoxide anion (O_2_−), hydrogen peroxide (H_2_O_2_), hydroxyl radicals (OH) (Kumar and Adki, 2018). Antibiotics like azithromycin, doxycycline, tigecycline, pyrvinium pamoate, chloramphenicol, 17-allylaminogeldanamycin, methotrexate, and anthracycline are principally used in the treatment of bacterial infections and manifest anticancer activity by inhibiting the cell growth on cell lines like MCF7, T47D, etc. at the concentration range of 50–200 nM.

Anti-inflammatory agents also act as anticancer agents by inhibiting various inflammatory mediators like TNF-α, IL2 IL12, transforming growth factor beta (TGF-β), etc. Scientists have reported that COX2 and EGFR signaling is quite common to both cancer and inflammation, so COX2 inhibitors and the combination of both COX2 and EGFR inhibitors are potential anticancer agents at lower doses. COX2 enzyme has a crucial role in tumor growth (Rayburn et al., 2009). Various natural alkaloids comprising indole, quinoline, isoquinoline, and piperidine manifests COX-2 inhibition (Figure 5). Pyrano-quinolone analogs also inhibit TNF-α and IL6 which are also pro-inflammatory mediators. Therefore, from the above discussion, it is clear that anti-inflammatory agents also act as anticancer agents (Dey et al., 2020).

Naturally occurring anti-mitotic agents like Nocodazole, Taxol, AZ138, BPR0L075, Vinblastine, Taxanes, and Epothilones, act by inhibition of PI3K and APC and RB pathways, and microtubule destabilizing agent which is related to the anticancer activity (Dall’Acqua, 2014). Dolastatin-10, Aplidine, Halichondrine-B, and Descodermolide inhibit the microtubule growth, ultimately, acting as anti-cancer agents. Therefore, from the above discussion, the naturally occurring compounds act by multi-targeting anticancer agents (Kumar and Adki, 2018).

Taylor et al. reported a rare natural product, Deacetylnemorone member of the diterpenoid family, as an anticancer agent which inhibits cell growth on different cancer cell lines (Taylor et al., 2019). Deacetylnemorone acts by resensitizing chemotherapy resistance of cancer. Activity reported for Deacetylnemorone is anti-angiogenic and cancer cell growth inhibitor. Curcumin was reported as a natural active constituent used in the treatment of pancreatic cancer by the multitarget approach. Curcumin inhibited various targets like NF-κB, miR-221, COX-2, and their effectors such as PTEN, p27, p57, and pro-inflammatory cytokines which ultimately causes cancer cell growth and progression (Sahebkar, 2016). Curcumin also inhibits phosphorylation STAT-1, STAT-3, and Notch signaling pathways which are responsible for pancreatic cancer cell growth. Reports also portray that curcumin is effective and selective towards different pancreatic cancer cell lines namely MiaPaCa-2, Panc-1, AsPC-1, BxPC-3, and Pan02.

Diederich et al. reported curcumin as a multitarget natural compound with its application in cancer interruption and therapy. Curcumin modulates numerous molecular targets and blocks signaling tracks such as COX-2, STAT, NF- κB, VEGFR, EGFR, P13/Akt, and mTOR. It also influences cell cycle regulators like CDK, B-catenin, Tcf-4 for cancer progression (Teiten et al., 2010; Teiten et al., 2014). Curcumin activates the cancer cell death proteins, namely PARP, Bcl-2, Bcl-xL, LC-3II, and cyclin-B1. Curcumin and its analogs also act against diverse cancers like multiple myeloma, rectal cancer, pancreatic cancer, osteosarcoma, colon neoplasm, and colorectal cancer. Hence, curcumin is a multitargeting agent in cancer mitigation (Teiten et al., 2010).

Another study reported piperine as an anticancer agent which acts on various targets such as NF-κB, c-Fos, cAMP response element-binding protein (CREB), activated transcription factor 2 (ATF-2), and proinflammatory cytokine gene expression in B16F-10 melanoma cells. Piperine inhibited collagen matrix at a concentration of 2.5, 5, and 10 μg/ml against B16F-10 melanoma cells at a dose-dependent analysis (Pradeep and Kuttan, 2004). It also inhibited matrix metalloproteases with the aid of the zymographic method. Piperine restrained nuclear translocation of p65, p50, c-Rel subunits of NF-κB and other transcription factors such as ATF-2, c-Fos, and CREB that resulted in cancer growth inhibition (Pradeep and Kuttan, 2004).

## Theranostics Perspective of Natural Anticancer Compounds

Theranostics is the combination of diagnosis and treatments. It is a therapy in which a combination of one radioactive drug will diagnose the tumor and other radioactive drugs will treat the main tumor and metastatic tumors (Silva et al., 2019). This combination will diagnose cancer at various locations effectively and monitor the progress of the disease and guide for another treatment of chemotherapy or surgery whichever may be needed depending on the disease condition (Garofalo et al., 2020).

For many years, natural products have been used for their diverse chemical structures and unique targeted activities. Natural products are easily compatible with the human body and display low toxicity. Many natural products including porphyrins, perylene quinone derivatives, curcumin are photosensitizers and sono-sensitizers, which have been widely applied in fluorescence imaging, diagnosis, photodynamic therapy (PDT), and sonodynamic therapy (SDT) (Cova et al., 2019). In PDT, excited photosensitizers oxidize cellular macromolecules like nucleic acids and proteins, resulting in tumour cell apoptosis by producing reactive oxygen species. In SDT, ultrasound wave interacts with the water molecules in the environment, causing ultrasonic cavitation during which tiny cavities nucleate, grow, and collapse (Ma et al., 2019). Theranostic agents like porphyrin and its derivatives are approved for therapeutic usage in cancer management as they have lower toxicity and appropriate biocompatibility. Hematoporphyrin derivatives are photosensitizers, approved for clinical PDT. Porphyrinoid biohybrid materials are approved for phototheranostics. The light sensitivity of curcumin and its derivatives is weaker in the therapeutic window of wavelength, that limits their role as theranostic agents (de Araújo et al., 2020).

The theranostic role of SDT is only to tumorstastic action as it only inhibits the tumor growth especially of solid tumors. On the other hand, PDT has “-cidal effect” on the tumors. It is interesting to note here that to date, there is no SDT approved for the medicinal use however several photosensitizers are used for cancer management as a part of photodymanic therapy. The exact mechanism behind the SDT needs to be revealed soon, and natural SDT with better safety and efficacy in cancer management must be produced (Sharma et al., 2017).

Some of the examples of natural products used as theranostic agents are as follows:

• Porphyrin
• Perylene quinine (Hypocrellin, Hypericin, Cercosporin, Elsinochrome)
• Cercosporin
• Elsinochrome
• Curcumin
• Pheophytin
• Psoralens
• Berberine chloride
• Graphene

Porphyrin, as a theranostic agent, has photodynamic, sonodynamic, and radiotherapeutic roles in the treatment of cancer. It is also used as a diagnostic agent for fluorescence imaging, magnetic resonance imaging, and photoacoustic imaging. The use of porphyrin is limited as a theranostic agent due to its poor selectivity for tumor cells. When nanoparticles are used as carriers of porphyrins, their anticancer effect improves. Perylene quinone has many pigments which show chemical and biological properties, making them diagnostic and therapeutic agents in PDT and SDT. Some pigments such as hypocrellins, elsinochrome, hypericin, cercosporin are included in perylenequinones. Hypocrellin, a pigment of perylenequinone category, is extracted and isolated from the *Hypocrella bambusae*, which is parasitic on Fargesia plant, has absorption between 400–800 nm, and has a high oxygen species yield, making it suitable for the treatment of tumors. It is less toxic and has a fast metabolism rate *in vivo*, and it is one of the best-known new generation phototheronostic agents that has potential for the development of drugs (Bisen, 2016; Peyvandipour et al., 2018).

Cercosporin is a photodynamic photosensitive pereylene quinoline derivative that is often used with a co-polymer to target carcinogenic cells. Polymer releases cercosporin in acidic conditions and offers cancer treatment for patients. Elsinochrome is a theranostic agent showing photodynamic property which is used for targeted drug delivery to cancerous cells (Wilken et al., 2011).

Pheophytin, a form of seagrass, is a natural anticancer pigment that is evaluated in the management of adenocarcinoma A549 cells. Photo-reduction of pheophytin has been observed in various mixtures containing PSII reaction centers. Similarly, Psoralens (furanocoumarins) are explored for the treatment of certain lymphomas as targeted therapeutics in conjunction with ultraviolet rays (Shailaja et al., 2019).

Berberine chloride is an orally bioavailable, hydrochloride salt form of berberine, a quaternary ammonium salt of an isoquinoline alkaloid and active component of various Chinese herbs (*Coptis chinensis* French*, Coptis deltoidea* C. Y. Cheng et Hsiao *and Coptis teetoides* C. Y. Cheng) (Neag et al., 2018), with good anticancer, photodynamic, anti-inflammatory, and anti-lipidemic activities (Belwal et al., 2020). Berberines are isoquinoline derivatives and belong to protoberberines alkaloids. Berberine has been shown to have a major hormetic dose-response, where a low dose actively promotes the development of cancer cells whereas a high dose serves as an anticancer agent. Furthermore, because of its widespread presence in numerous plant species and low toxicity, berberine hydrochloride has the potential to be a powerful anticancer agent in the future (Singh and Sharma, 2018).

Graphene is an inorganic material that is used for making nanocomposites for drug delivery due to its lower toxicity and additional anticancer action (Hoseini-Ghahfarokhi et al., 2020). Apart from being an anticancer agent, the graphene is also has photodynamic action (Yang et al., 2019). Graphene is generally used as a nanocarrier for drug delivery, especially anticancer drug delivery (Jampilek and Kralova, 2021). The graphene nanocomposites have better drug loading and protective action, targeted delivery, are suitable for theranostic role, and have effective photodynamic action. It is used as a theranostic agent due to its anticancer action (Rosli et al., 2019).

## Natural Anticancer Compounds for Personalized Medicine

There is substantial increase in our understanding of health and disease related aspects of human life due to technological advancement and multiomics approaches. To achieve proper effectiveness, the use of personalized pairings of precision targeted drugs recognized by proteogenomics will necessitate specialized modelling based on the latest methodologies (Li and Bergan, 2020). Personalized medicine is a domain which aims to develop therapeutics for a single subject or group of subjects derived from current and past data capturing of physiological health and the environment exposure. Precision oncology has demonstrated some significant success in the last decade, despite the fact that it is expensive (Cutler, 2020). Consequently, the precision medicine approach, which was endorsed in 2015, has propelled the personalized medicine forward by necessitating the FDA to design new technologies for evaluating personalized medicine (Krzyszczyk et al., 2018).

Well over 700 natural compounds have now been confirmed to have pharmacological function, with many of them capable of targeting cellular processes or deregulated genes that inhibit tumorigenesis (Mazumder et al., 2018). For every cancer patient or group of patients, there is unique genetic makeup that serves as a cancer operator and can shift throughout therapy to stimulate response processes. When it is used in conjunction with certain drugs, several natural compounds with established molecular targets demonstrated good therapeutic benefits by restricting signalling proteins that facilitate tumor progression (Cerella et al., 2015). The continuously advancing domain of immunooncology has improved our understanding of tumor-specific immune responses as well as the ability of targeted chemotherapy drugs to stimulate the anti - tumor immunity toward carcinoma. It was recently noted that targeted therapies like oxaliplatin, that also kill cancer cells by triggering a host immune system, can make tumours more susceptible to checkpoint blockade treatment (Pfirschke et al., 2016). The distribution of key anti-apoptotic Bcl-2 proteins forecasted a complementary response (Mazumder et al., 2018). The brominated alkaloid isofistularin-3, derived from the marine sponge *Aplysina aerophoba*, is shown to suppress DNA methyltransferase (DNMT1) (Florean et al., 2016; Mazumder et al., 2018). In RAJI and U937 cells, isofistularin-3 combined with tumour necrosis factor related apoptosis inducing ligand (TRAIL) demonstrated strong synergy (Florean et al., 2016; Mazumder et al., 2018). Recently, it was observed that combining ursolic acid, curcumin, and resveratrol to locate STAT3, mTORC1, and AMPK action substantially lowered prostate cell proliferation and attenuated glutamine metabolism, thereby attacking a critical true sign of cellular kinetics (Kuntz et al., 2017).

Taken together, the important development of identified targeted agents of natural origin, as well as the immunogenic capability of such agents when combined with existing drugs, will contribute to future pharmacotherapy opportunities for tumour targeting.

## Conclusion and Future Prospects

For a long time, natural phytochemicals have been proven to be effective against different types of cancer. The opportunities and prospects of natural products for drug discovery are being significantly extended with the exploration of plant endophytic fungi, which have been recognized as a decent source of certain bioactive metabolites having an anticancer activity (Chandra, 2012). Major natural anticancer compounds like camptothecin, taxol, vinca alkaloids, and podophyllotoxin have been obtained from an endophytic fungus. Marine-derived bioactive compounds also have a large potential to produce an anti-cancer effect (Abdelmohsen et al., 2014). Similarly, bioactive metabolites from insects have potential for drug discovery in the rapidly growing areas of research like microbial genomics through genome mining and metagenomics (Bachmann et al., 2014; Charlop-Powers et al., 2014).

Drug research and production would necessitate a close multidisciplinary partnership in the exploration of natural product leads using combinatorial and medicinal chemistry, complete synthesis, combinatorial biochemistry, and nanotechnology. Natural product analysis that combines nanotechnology and analytical methods is a dominant strategy for identifying biologically active substances having distinct structures as well as modes of activity. Given nature’s immeasurable diversity, it is obvious that chemical leads capable of interacting with all therapeutic targets can be produced, indicating a greater potential for the production of highly effective therapeutic agents. Several technological advancements in the drug discovery fields will lead to speeding up the process of finding suitable drug candidates of natural origin that have potential anticancer activity. The multitarget approach of drug discovery has provided an attractive niche for medicinal scientists as it reduces the burden of the multidrug regime for cancer management and also reduces the side effects associated with them. Cutting edge analytical tools and bioinformatics especially machine learning will help in the process of drug discovery to find out the suitable hits in the early drug discovery phase of natural anticancer discovery. Natural phytochemicals continue to be a valuable substitute of scaffolds with high structural diversity and diverse antitumor activity that can be established directly or used as starting points for modeling into new therapeutics. The theranostic potential of such natural compounds is immense and we will witness much future research towards such theranostic agents of natural origin.
